# Three-dimensional visualization measurement and analysis of human hepatic pedicle based on Laennec’s capsule

**DOI:** 10.3389/fsurg.2025.1598774

**Published:** 2025-05-16

**Authors:** Zhiyu Lin, Xin Xia, Yuchuan Luo, Huan Lei, Hongyin Liang, Hui Zhang, Zhiwei Jiang, Tao Wang

**Affiliations:** ^1^ Department of General Surgery (Hepatopancreatobiliary Surgery), The Affiliated Hospital of Southwest Medical University, Luzhou, Sichuan, China; ^2^ Department of General Surgery, The General Hospital of Western Theater Command, Chengdu, Sichuan, China

**Keywords:** Laennec's capsule, hepatic pedicle, three dimensional reconstruction, 3D printing, segmenation

## Abstract

**Background:**

Laennec's capsule serves as a crucial anatomical landmark in liver surgery, yet imaging-based morphometric studies of this fibrous membrane remain limited. This study introduces a novel 3D reconstruction technique to visualize and measure the hepatic pedicle via Laennec's capsule, with validation focused on the left portal branch and development of a 3D-printed model for surgical planning.

**Methods:**

30 patients undergoing left hemihepatectomy were recruited to validate the accuracy of a Laennec's capsule-based 3D reconstruction system (Hisense CAS). Preoperative measurements of the left branch length, diameter, and the angle between the main trunk and the hepatic pedicle were compared with intraoperative findings. Additionally, 100 adults without hilar lesions underwent 3D reconstruction for comprehensive morphological classification and statistical analysis of the hepatic pedicle trunk and main branches.

**Results:**

No statistically significant differences emerged between the 3D reconstruction data and intraoperative measurements, confirming the method's accuracy. Four distinct branching patterns were identified, with Type I accounting for 88% of cases. At the first hepatic hilum, the mean outer diameter of the hepatic pedicle was 24.10 ± 6.16 mm. The left and right main branches demonstrated considerable variability in length (20.59 ± 6.38 mm vs. 21.99 ± 7.97 mm) and outer diameter (18.04 ± 4.48 mm vs. 21.18 ± 4.23 mm). The angle between the left and right main branches was 140.81 ± 16.72°. Furthermore, a 3D-printed liver model was developed to aid in surgical planning and education.

**Conclusions:**

Three-dimensional reconstruction based on Laennec's capsule accurately reflects hepatic pedicle anatomy and its variations. The predominance of Type I branching underscores the need for precise classification during liver surgery. This approach provides valuable morphological data for individualized surgical planning, improves intraoperative safety, and sets the stage for further research integrating alternative imaging modalities and larger patient cohorts.

## Introduction

Anatomical hepatectomy remains the principal strategy for managing malignant liver tumors and other hepatic disorders, primarily due to its positive impact on improving long-term patient survival rates (1, 2). The approach has progressively gained recognition and acceptance in the field of hepatic surgery, especially with the emergence of laparoscopic techniques. Notably, laparoscopic anatomical hepatectomy hinges on two key components: selecting the optimal surgical approach and effectively controlling the hepatic inflow and outflow tracts (3). Consequently, the Glisson sheath technique, a cornerstone of anatomical hepatectomy, is widely employed in laparoscopic surgeries (4).

Among the various anatomical structures of the liver, Laennec's capsule has garnered increased attention in recent years. First identified by Laennec in 1,802 and later recognized by Couinaud (5), this continuous membrane envelops the entire liver surface, including the bare area, gallbladder bed, and vascular pedicles. Historically, surgeons overlooked its role in liver surgery for nearly two centuries, yet recent studies have highlighted its importance. Hayashi et al. (6) demonstrated that the outer membrane of the Glissonean pedicle originates from the surrounding Laennec's capsule rather than from the pedicle itself. Further investigations by Sugioka et al. (7) using Azan-Mallory staining confirmed that Laennec's capsule is a non-serosal membranous structure covering the entire liver parenchyma and the main trunks of the hepatic vein. Monden et al. (8) later identified two distinct layers of Laennec's capsule encasing the hepatic vein, illustrating its morphological complexity.

Despite this renewed emphasis on Laennec's capsule in laparoscopic liver surgery, most existing research has focused on histological rather than imaging aspects. Consequently, there is a paucity of structured imaging analyses examining the capsule's morphology and its relationship to the hepatic pedicle. Given Laennec's capsule's high anatomical specificity and clear visualization potential, an imaging-based investigation could significantly improve surgical planning and the precision of liver resections. Therefore, We aimed to perform 3D visualization and measurement of the hepatic pedicle, validate measurements against intraoperative findings of the left portal branch, and develop a 3D-printed model for clinical and educational use.

## Methods

### Study population and ethical approval

This study was conducted at The General Hospital of Western Theater Command PLA from January 1, 2021, to June 1, 2024, and was approved by the institutional ethics committee (license number: 2023EC5-ky005). Adult patients aged 18 to 65 years were considered eligible if they (1) showed no evidence of space-occupying lesions, biliary dilatation, or cirrhosis; (2) had no previous surgical history or anatomical variations affecting hepatic pedicle morphology; (3) had clear and complete contrast-enhanced CT scans demonstrating the main hepatic pedicle structures, including primary and secondary branches; and (4) consented to participate after review by the ethics board.

### Patient selection for validation cohort

The validation cohort consisted of 30 adult patients undergoing left hemihepatectomy, deliberately selected to standardize intraoperative measurements and focus on the left hepatic pedicle. This surgical procedure was chosen because it provides consistent anatomical exposure of the left portal branch, facilitating precise intraoperative measurements of its length, external diameter, and angle relative to the main hepatic trunk. These measurements were critical for validating the accuracy of the Laennec's capsule-based 3D reconstruction method by enabling direct comparisons with preoperative imaging data. The focus on left hemihepatectomy ensured methodological consistency and minimized variability in surgical access to the hepatic pedicle structures.

### Image acquisition and preprocessing

All participants underwent abdominal contrast-enhanced CT scanning using a 64-row multi-slice spiral CT scanner (GE LightSpeed VCT, GE, USA). The DICOM images were then imported into the Hisense Computer Assisted Surgery (CAS) system for 3D processing. Laennec's capsule was identified on contrast-enhanced CT as a thin, hypodense fibrous layer surrounding the hepatic pedicle, distinct from the hyperdense Glisson's sheath and adjacent parenchyma. Two senior radiologists independently annotated the capsule slice by slice, with inter-observer agreement assessed via Cohen's kappa (*κ* = 0.85). Measurements were performed twice by each radiologist, and averaged values were used for analysis. The grayscale values in each DICOM slice to Hounsfield units (HU) using the following formula:

HU=pixel_value*RescaleSlope+RescaleIntercept

Here, pixel_value denotes the gray value of each pixel in the original DICOM image, while *RescaleSlope* and *RescaleIntercept* are obtained from the DICOM image header file. Afterward, the window level was adjusted to −450 to 600 HU (window width 1,500–2,000 HU) for optimal liver structure visualization. Each labeled CT dataset was then cropped to a minimal cuboid region enclosing the liver parenchyma, and all data (CT images and manual labels) were resampled using third-order spline interpolation to standardize voxel size (1.0 mm × 0.75 mm × 0.75 mm recommended).

### Segmentation network

A 3D U-net deep neural network was applied to segment the hepatic pedicle. Data augmentation steps—including angle rotation, grayscale adjustments, and flipping—were performed to enhance the network's generalizability. The training process used a combined cross-entropy and Dice coefficient loss function, defined as:

$$\mathrm{Loss}\;\;\mathrm{Function}=L_{Cross-Entropy}\;+\;L_{Dice}=\;-\overset{N}{\underset{n=1}{\sum }}\;y_{i}\log\;(\hat{y_{i}})\;-\;\frac{1}{\left|N\right|}\underset{n\in N}{\sum }\frac{2\sum y_{i}\hat{y_{i}}}{\sum (y_{i}+\hat{y_{i}})}$$

Where *y_i_* and *ŷ* represent the standard and predicted values for pixel *i*, respectively. The Ranger optimizer, which merges RAdam and LookAhead, was used, and the initial learning rate of 1 × 10^−4^ employed a warmup strategy. After training, the network automatically segmented the primary, secondary, and tertiary branches of the hepatic pedicle, from which the system extracted measurements of length, external diameter, and branch angles.

### Operative measurements

Between January and June 2024, a separate cohort of 30 adult patients (16 females and 14 males; age range 34–86 years) underwent left hemihepatectomy for various liver conditions (13 Intrahepatic Bile Duct Stone, 12 hepatocellular carcinomas, 4 hepatic hemangiomas and 1 Cirrhotic Nodular Hyperplasia). Preoperatively, contrast-enhanced CT scans were acquired and processed for 3D reconstruction using the same protocol described above.

Intraoperatively, the first hepatic hilum was fully exposed by dissecting along the plane between Laennec's capsule and the Glisson pedicle. The “first hepatic hilum” refers to the initial bifurcation point of the hepatic pedicle into left and right main branches. The length and external diameter of the left branch (from its origin to the third hepatic portal) and the angle between the left branch and the main hepatic trunk were measured with a probe. The “third hepatic portal” denotes the tertiary branching point within the intrahepatic pedicle. These intraoperative measurements were then compared with the corresponding preoperative 3D reconstruction data to evaluate the accuracy of the imaging-based method.

### Statistical analysis

All statistical analyses were conducted using SPSS 25.0 software (IBM Corp., Armonk, NY, USA). Continuous variables are presented as mean ± standard deviation (*x¯* *±* *s*), whereas categorical variables are expressed as percentages (%). A paired *t*-test was used to compare the imaging-based measurements with those obtained intraoperatively, and values of *P* *<* *0.05* were considered statistically significant.

## Results

### Validation of the 3D reconstruction

**Figure 1:**
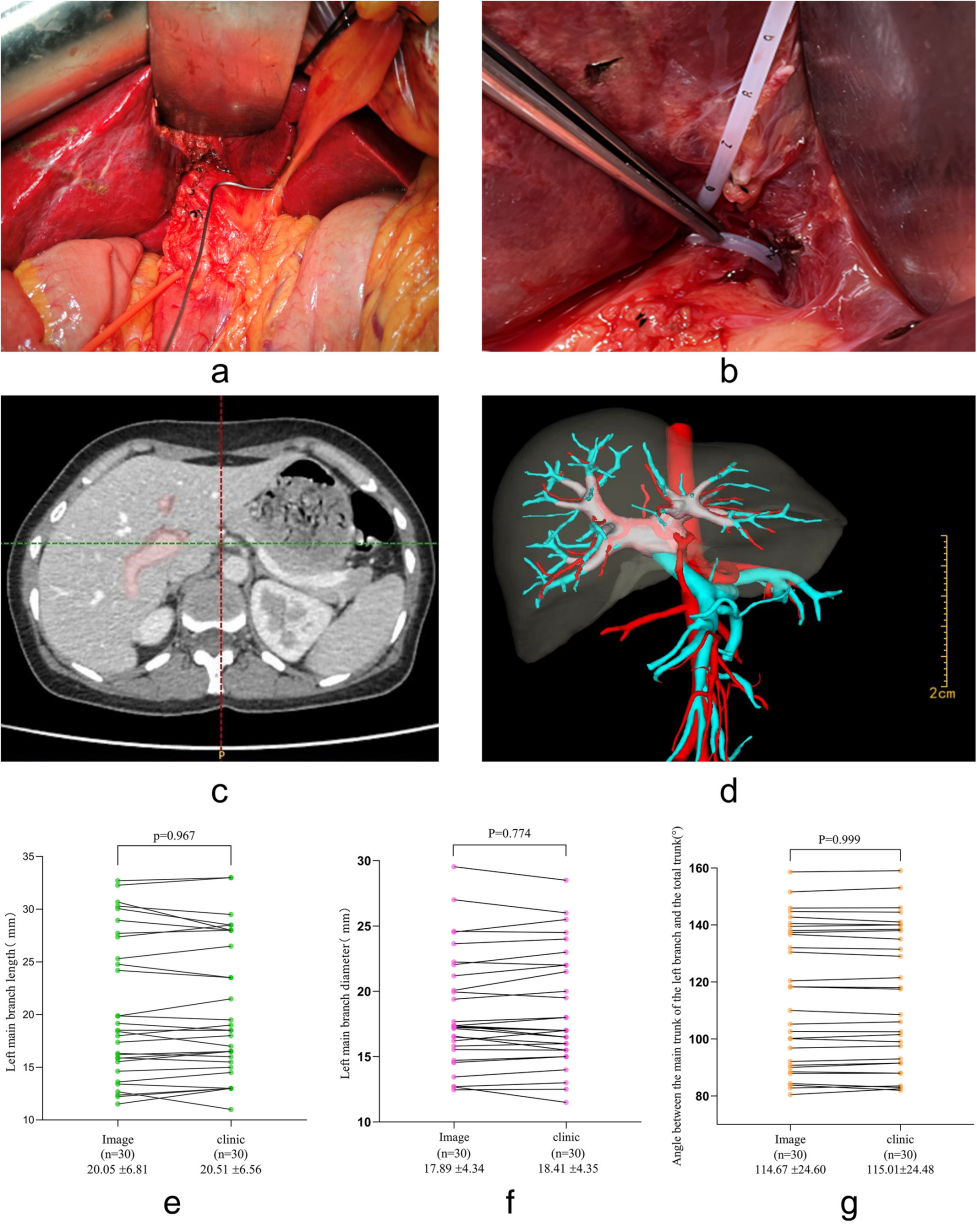
Comparisons between clinical and imaging data demonstrating the accuracy of the 3D reconstruction method.b. **(a)** Intraoperative measurement of the angle between the main hepatic pedicle and the left main branch. **(b)** Intraoperative measurement of the external diameter of the left main branch. **(c)** Workflow diagram illustrating 3D reconstruction of the hepatic pedicle based on Laennec's capsule. **(d)** The reconstructed 3D model, with the pink region representing the hepatic pedicle derived from Laennec's capsule. **(e)** Paired *t*-test analysis comparing the length of the left main branch between imaging and clinical measurements. **(f)** Paired *t*-test analysis for the external diameter of the left main branch. **(g)** Paired *t*-test analysis for the angle between the main hepatic pedicle and the left main branch.

A total of 30 patients (17 females, 13 males; age range 56–75 years) who underwent left hemihepatectomy were used to validate the accuracy of the three-dimensional (3D) reconstruction based on Laennec's capsule. The demographic and clinical characteristics of this cohort are summarized in Table 1, including gender, age, diagnosis, pathological type, and comorbid diseases, providing a comprehensive overview of the study population. Intraoperatively, the hepatic pedicle and its left branch were dissected (Figures 1a,b), and the length and external diameter of the left main branch, as well as the angle between the left branch and the main trunk, were measured. Comparison of these operative findings with the corresponding 3D reconstruction data (Figures 1c,d) showed no significant differences (Figures 1e–g). Specifically, the mean left branch length measured 20.05 ± 6.81 mm via imaging vs. 20.51 ± 6.56 mm intraoperatively (*P* = 0.067), while the external diameter was 17.89 ± 4.34 mm on imaging vs. 18.41 ± 4.35 mm in surgery (*P* = 0.774). The angle between the left main branch and the hepatic trunk showed a similar lack of difference between imaging (114.67 ± 24.60°) and operative (115.01 ± 24.48°) measurements (*P* = 0.999). These findings confirm that the 3D reconstruction method based on Laennec's capsule accurately reflects clinical anatomy.

**Table 1:** Demographic and clinical characteristics of the validation cohort, detailing age, sex, pathology, and comorbidities.

Patient	Gender	Age	Diagnosis	Pathological type	Comorbid diseases
1	W	60	Liver Cancer	CRLM	none
2	W	83	Liver Cancer	CCA	EH
3	W	50	Hepatolithiasis	IHBD stone	EH, T2DM
4	M	55	Liver Cancer	HCC	CHB
5	M	61	HH	CH	EH
6	M	67	Cirrhosis of the liver	CNH	CHB
7	W	43	HH	CH	EH
8	M	61	HH	CH	EH
9	M	55	Liver Cancer	HCC	CHB
10	M	74	Liver Cancer	HCC	EH, CHB
11	W	36	Liver Cancer	HCC	CHB
12	M	69	Liver Cancer	HCC	EH, CHB
13	M	59	Liver Cancer	HCC	CHB
14	W	54	HH	CH	SLC
15	M	66	Liver Cancer	CCA	EH
16	M	71	Liver Cancer	HCC	CHB
17	W	72	Hepatolithiasis	IHBD stone	EH
18	W	74	Hepatolithiasis	IHBD stone	none
19	W	66	Hepatolithiasis	IHBD stone	none
20	M	79	Hepatolithiasis	IHBD stone	none
21	W	52	Hepatolithiasis	IHBD stone	none
22	M	60	Liver Cancer	HCC	CHB
23	W	67	Hepatolithiasis	IHBD stone	none
24	W	53	Hepatolithiasis	IHBD stone	none
25	W	34	Hepatolithiasis	IHBD stone	HH
26	M	54	Liver Cancer	HCC	CHB
27	M	57	Hepatolithiasis	IHBD stone	SLC
28	W	73	Hepatolithiasis	IHBD stone	EH
29	W	71	Hepatolithiasis	IHBD stone	none
30	W	55	Hepatolithiasis	IHBD stone	none

Abbreviations: CRLM, colorectal liver metastases; CCA, cholangiocarcinoma; EH, essential hypertension; IHBD stone, intrahepatic bile duct stone); T2DM, type 2 diabetes mellitus; HCC, hepatocellular carcinoma; CHB, chronic hepatitis B; HH, hepatic hemangioma; CH, cavernous hemangioma; CNH, cirrhotic nodular hyperplasia; SLC, simple liver cyst.

### Performance of the 3D U-Net segmentation algorithm

The 3D U-Net was validated on the dataset to assess its performance in segmenting liver and tumor structures. The algorithm achieved a mean Dice Similarity Coefficient (DSC) of 0.984 for liver segmentation and 0.840 for tumor segmentation, outperforming the U-Net baseline method. These results highlight the robustness of the 3D U-Net in accurately delineating hepatic structures, particularly the liver parenchyma, though tumor segmentation presented greater challenges due to its morphological variability. The annotation process for the hepatic pedicle involved initial labeling by personnel with professional imaging knowledge, followed by confirmation from a specialist doctor, ensuring a rigorous review process to enhance the reliability of the segmented data used for subsequent measurements.

### Morphological classification of hepatic pedicles

**Figure 2:**
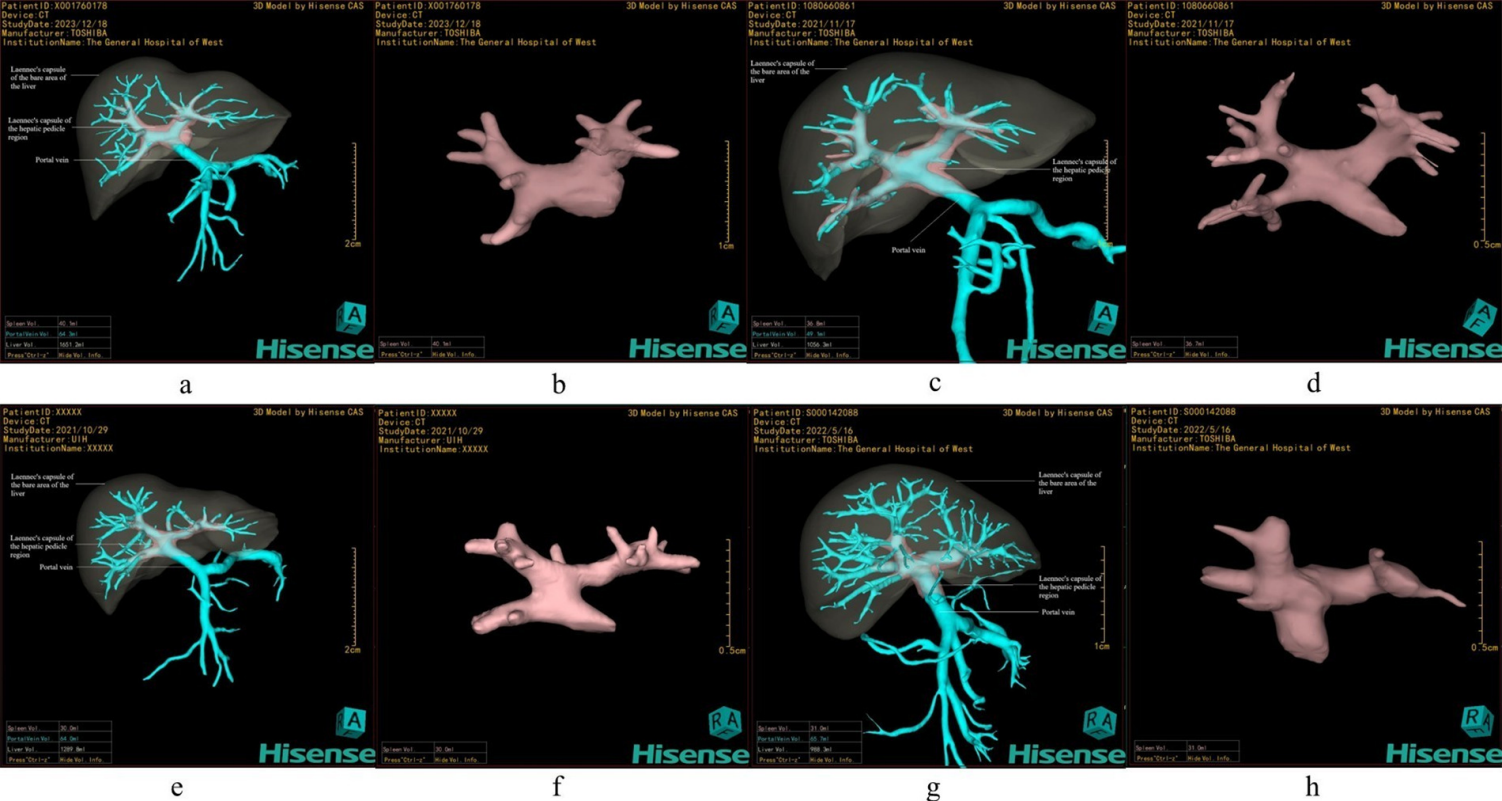
Classification of hepatic pedicle branching patterns based on Laennec's capsule. **(a,b) Type I**: Classic bifurcation into left and right main branches. **(c,d) Type II**: Three-branch type without a true right main trunk, dividing into left, right anterior, and right posterior branches. **(e,f) Type III**: The right anterior branch originates from the left main branch. **(g,h) Type IV**: A special variant distinct from the aforementioned three types.

Among 100 adult livers reconstructed by the same 3D method, four distinct branching patterns were identified based on the main trunk, left branch, and right branch configuration (Figure 2).

• Type I (Figures 2a,b): Classic bifurcation into left and right main branches, observed in 88% (88/100) of cases.
• Type II (Figures 2c,d): A three-branch pattern without a true right main trunk, occurring in 5% (5/100) of cases.
• Type III (Figures 2e,f): The right anterior lobe branch arises from the left branch, also observed in 5% (5/100) of cases.
• Type IV (Figures 2g,h): A rare variant comprising 2% (2/100) of cases, distinct from the previous three patterns.

### Morphometric measurements of hepatic pedicles

Table 2 summarizes the length and external diameter of the main trunk, left main branch, right main branch, and their secondary divisions in the 100 reconstructed specimens. At the first hepatic hilum, the main hepatic pedicle's mean outer diameter was 24.10 ± 6.16 mm. The left main branch measured 20.59 ± 6.38 mm in length and 18.04 ± 4.48 mm in outer diameter, whereas the right main branch was 21.99 ± 7.98 mm long and 21.18 ± 4.23 mm in outer diameter. For the two major divisions of the right main branch (the right anterior and posterior lobar branches), both length and diameter also exhibited inter-individual variability.

**Table 2:** Length and outer diameter of 100 human hepatic pedicles based on Laennec's capsule 3D reconstruction (mm).

Project	Part	Mean ± SD	SE
Main branch	OD	24.10 ± 6.16	0.69
Left main branch	Length	20.59 ± 6.38	0.72
	OD	18.04 ± 4.48	0.5
Right main branch	Length	21.99 ± 7.98	0.97
	OD	21.18 ± 4.23	0.52
The main right anterior branch	Length	13.20 ± 5.43	0.68
	OD	15.25 ± 3.39	0.39
The main right posterior branch	Length	12.03 ± 4.13	0.63
	OD	13.01 ± 2.80	0.4

OD, outside diameter; Mean ± SD, mean ± standard; SE, standard error.

The angular relationships are presented in Table 3. In Type I livers (the majority subtype), the mean angle between the hepatic pedicle and the left main branch (ALM) was 114.35 ± 22.91°, and between the pedicle and the right main branch (ARM) was 140.81 ± 16.72°. The angle formed by the left and right main branches (ALR) was 142.63 ± 15.66°. These measurements highlight the considerable anatomical variability within the hepatic hilum, reinforcing the need for careful preoperative evaluation.

**Table 3:** Angle of 100 human hepatic pedicles based on Laennec's capsule 3D reconstruction (°).

Project	Mean ± SD	SE
ALM	114.35 ± 22.91	2.61
ARM	140.81 ± 16.72	2.49
ALR	142.63 ± 15.66	1.89

ALM, angle between left main branch and main branch; ARM, angle between right main branch and main branch; ALR, angle between left main branch and right main branch; Mean ± SD, mean ± standard deviation, SE, standard error.

### Three-dimensional printing of a representative liver model

**Figure 3:**
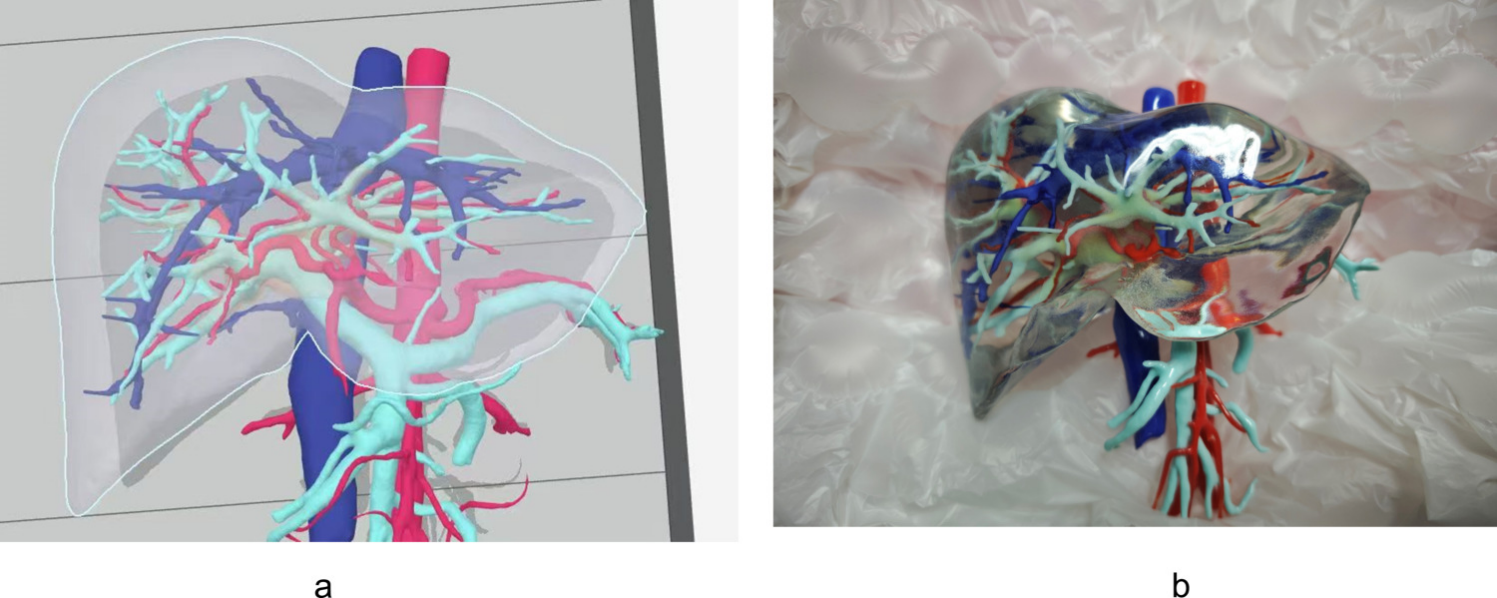
A novel 3D-printed liver model derived from Laennec's capsule-based reconstruction. This model offers an intuitive representation of the hepatic pedicle and surrounding anatomical structures, enhancing preoperative planning, surgical simulation, and educational applications. **(a)** 3D reconstruction model, in which red is the artery, dark blue is the vein, light blue is the portal vein system, and pink region representing the hepatic pedicle derived from Laennec's capsule. **(b)** 3D printed model.

Based on the statistical analysis of Type I livers, which constituted nearly 90% of cases, a representative 3D liver model was designed (Figure 3a). This model was then printed using a transparent resin to illustrate the main vascular structures within the Laennec's capsule (Figure 3b). The resulting physical model proved valuable for preoperative planning, intraoperative orientation, and educational demonstrations, offering a highly detailed depiction of the hepatic pedicle and surrounding anatomy.

## Discussion

Over the past decade, an increasing number of surgeons have recognized the importance of Laennec's capsule in laparoscopic liver resection, aiming for a standardized surgical approach that leverages its unique anatomy (7, 9, 10). The present study represents a novel step forward by integrating Laennec's capsule into three-dimensional (3D) visualization and morphometric analysis of the human hepatic pedicle. We report the first instance of 3D reconstruction of this fibrous membranous structure through preoperative imaging, thereby expanding the scope of research on Laennec's capsule beyond histological observations. Our key findings encompass four main points: (1) 3D reconstruction data from Laennec's capsule strongly correlate with intraoperative measurements, confirming the reliability of imaging-based assessments; (2) hepatic pedicles exhibit several distinct branching types, highlighting the anatomical variability critical for surgical planning; (3) imaging measurements of the hepatic pedicle's length, diameter, and angles underscore the value of precise morphological data for tailored resections; and (4) we successfully created and 3D printed a representative liver model, offering potential benefits in surgical simulation and education.

Laennec's capsule itself, being predominantly composed of elastic and collagen fibers, adheres to the liver parenchyma while remaining independent of both the serosal layer and the Glisson's sheath (6, 7). This anatomical gap provides surgeons with a reliable and reproducible plane for hepatic pedicle and venous dissection (8, 11). In accordance with prior studies, these imaging data enhance our anatomical understanding of the liver, facilitating the construction of a comprehensive three-dimensional liver model (12). Furthermore, 3D reconstruction methods enable detailed visualization of vasculature, tumor distribution, and connective tissue interfaces, thereby contributing to safer intraoperative maneuvers and optimized preservation of healthy liver tissue (13, 14). Our Type I model, accounting for roughly 90% of observed cases, was transformed into a solid 3D-printed structure, assisting in preoperative planning, surgical navigation, and patient education.

Previous investigations have indicated the added benefits of Laennec's capsule-based dissections, particularly in laparoscopic techniques (15, 16). Combining this approach with ICG fluorescence imaging and robotic surgical systems has been shown to further improve surgical precision and reduce operative risks (17, 18). By classifying hepatic pedicle configurations and providing 3D morphological data, our study contributes to standardizing anatomic liver resections and establishing safety benchmarks for hepatobiliary procedures (19). These advances are expected to improve postoperative recovery, patient quality of life, and long-term clinical outcomes. However, several limitations need to be addressed. First, our validation of the 3D reconstruction relied on a relatively small sample of patients undergoing left hemihepatectomy. The inclusion of more varied surgical procedures and larger patient cohorts would increase the generalizability of these findings. Second, our analysis predominantly used contrast-enhanced CT data. While contrast-enhanced CT is widely used, its limited resolution for fibrous structures like Laennec's capsule may pose challenges, and MRI could offer enhanced soft-tissue contrast, as noted in prior studies (20). In patients with distorted anatomy, such as those with cirrhosis or tumor-related deformation, altered tissue planes and vascular distortion may affect segmentation accuracy. Future investigations that incorporate MRI or combine multiple imaging modalities may yield more comprehensive insights. Lastly, while our 3D-printed models hold promise for clinical and educational applications, further studies are warranted to evaluate cost-effectiveness, ease of incorporation into routine practice, and the impact on surgical outcomes.

## Conclusion

In summary, this study underscores the feasibility and clinical value of 3D visualization and measurement of the hepatic pedicle based on Laennec's capsule. By validating the consistency between preoperative reconstructions and intraoperative findings, we have demonstrated an effective framework for surgical planning, simulation, and navigation. Moreover, the production of a 3D-printed liver model enhances training opportunities and patient communication. With further refinements—such as the inclusion of MRI-based imaging and broader patient populations—this approach has the potential to advance both the safety and precision of minimally invasive liver surgery.

## Data Availability

The raw data supporting the conclusions of this article will be made available by the authors, without undue reservation.
